# National Prevalence and Effects of Multiple Chemical Sensitivities

**DOI:** 10.1097/JOM.0000000000001272

**Published:** 2018-01-12

**Authors:** Anne Steinemann

**Affiliations:** Department of Infrastructure Engineering, Melbourne School of Engineering, The University of Melbourne, Melbourne, Victoria, Australia; College of Science and Engineering, James Cook University, Townsville, Queensland, Australia; and Climate, Atmospheric Sciences, and Physical Oceanography, Scripps Institution of Oceanography, University of California, San Diego, La Jolla, California.

**Keywords:** asthma, chemical sensitivity, fragrance, MCS, multiple chemical sensitivities

## Abstract

**Objective:**

The aim of this study was to assess the prevalence of multiple chemical sensitivities (MCS), its co-occurrence with asthma and fragrance sensitivity, and effects from exposure to fragranced consumer products.

**Methods:**

A nationally representative cross-sectional population-based sample of adult Americans (*n* = 1137) was surveyed in June 2016.

**Results:**

Among the population, 12.8% report medically diagnosed MCS and 25.9% report chemical sensitivity. Of those with MCS, 86.2% experience health problems, such as migraine headaches, when exposed to fragranced consumer products; 71.0% are asthmatic; 70.3% cannot access places that use fragranced products such as air fresheners; and 60.7% lost workdays or a job in the past year due to fragranced products in the workplace.

**Conclusion:**

Prevalence of diagnosed MCS has increased over 300%, and self-reported chemical sensitivity over 200%, in the past decade. Reducing exposure to fragranced products could help reduce adverse health and societal effects.

## BACKGROUND

Multiple chemical sensitivities (MCS) is a medical condition characterized by adverse health effects from exposure to common chemicals and pollutants, from products such as pesticides, new carpet and paint, renovation materials, diesel exhaust, cleaning supplies, perfume, scented laundry products, and air fresheners.^1,2^ MCS can cause a range of acute, chronic, multiorgan, and disabling health effects, such as headaches, dizziness, cognitive impairment, breathing difficulties, heart palpitations, nausea, mucous membrane irritation, and asthma attacks.^3^ Individuals with MCS may not receive a diagnosis but nonetheless exhibit the condition of chemical sensitivity. Previous studies have found that MCS often co-occurs with asthma,^4^ as well as fragrance sensitivity,^5^ characterized by adverse health effects from exposure to fragranced consumer products.^6^

While MCS is perhaps the most common term, the condition is also known by other terms, such as chemical intolerance or environmental illness (specific to chemical exposures).^3^ MCS follows a two-step process of (i) initiation of the disease, often from exposure to petrochemical products, and then (ii) triggering of symptoms when exposed to problematic chemicals, often at low levels.^3,7^ While significant efforts have been devoted to developing case definitions and diagnostic criteria,^3,8,9^ a single internationally agreed-upon standard for prevalence studies is not yet established. Nonetheless, prior population-based studies of MCS, using specific and often different definitions and criteria, offer useful data on the extent and severity of the condition.

In the USA, two previous nationally representative studies, conducted in 2002 to 2003^4^ and 2005 to 2006,^5^ investigated the prevalence of MCS by using the key question developed by the California Department of Health Services (CDHS)^10^: “Compared to other people, do you consider yourself allergic or unusually sensitive to everyday chemicals like those in household cleaning products, paints, perfumes, detergents, insect spray and things like that?” This criterion reflects self-reported chemical sensitivity. To ascertain a medical diagnosis of MCS, the survey asked, “Has a doctor or health care professional ever told you that you have multiple chemical sensitivities?” This criterion reflects medically diagnosed MCS. These two USA studies found (respectively) a prevalence of 11.1% and 11.6% self-reported chemical sensitivity and 2.5% and 3.9% medically diagnosed MCS.

At the state and regional level in the USA, using the CDHS criteria, a survey of 4046 Californians in 1995^10^ found a prevalence of 15.9% self-reported chemical sensitivity and 6.3% medically diagnosed MCS. A survey of 1583 metropolitan Atlantans in 1999 to 2000,^1^ also using the CDHS criteria, found a prevalence of 12.6% self-reported chemical sensitivity and 3.1% diagnosed MCS. A survey of 1027 individuals in North Carolina in 1993,^7^ using a question similar to CDHS, found a prevalence of 33% chemical sensitivity.

In Sweden, using the chemical sensitivity scale for sensory hyperreactivity (CSS-SHR),^11^ an investigation of 1387 adults in Skövde found a prevalence of 33% of self-reported general odor intolerance, or being bothered by strong or pungent odors, such as perfume, cleaning agents, or flower scents.^12^ Also in Sweden, a survey of 3406 adult respondents from Västerbotten found 12.2% reported chemical intolerance to odorous pungent chemicals, such as perfumes and cleaning agents, and 3.3% were physician-diagnosed with chemical intolerance.

In Australia, a population-based survey of 4009 adults in South Australia in 2001 to 2002,^13^ using a variation of the CDHS question, found a prevalence of 15.9% of self-reported chemical sensitivity and 1% medically diagnosed MCS. In Japan, a national survey of 7245 adults,^14^ using the Quick Environmental Exposure and Sensitivity Inventory (QEESI) questionnaire,^9^ found a prevalence of 7.5% for chemical intolerance. In Korea, a survey of 379 adults, also using the QEESI, found a prevalence of 16.4% for chemical intolerance.^15^

While these studies provide useful context, we lack recent nationally representative data in the USA. A primary objective of this study is to provide a current estimate of the prevalence of MCS in the American population. Also, given previous studies indicating connections, a second objective is to investigate the co-occurrence of MCS with asthma and with fragrance sensitivity. Finally, because fragranced products are a common trigger, a third objective is to investigate the effects of exposure to fragranced products for individuals with MCS, which points to ways to reduce potential adverse effects.

## METHODS

To assess the prevalence and effects of MCS, an online survey was conducted with a random national cross-sectional sample of the adult US population, representative of age, gender, and region (*n* = 1137, 95% confidence level, 3% margin of error), drawn from a large national panel (over 5,000,000 people) held by Survey Sampling International. The survey instrument was developed and tested over a 2-year period before full implementation in June 2016. Response rate was 95%, and all responses were anonymous. (Details on survey methodology, questions, and data are provided in the files “Survey Methodology” and “Survey Data” as Supplemental Digital Content)

To promote comparability, the survey replicated questions from previous large US national, state, and regional MCS prevalence studies.^1,4,5,7,10^ In accordance, to ascertain medically diagnosed MCS, the survey asked, “Has a doctor or health care professional ever told you that you have multiple chemical sensitivities?” To ascertain self-reported chemical sensitivity, the survey asked, “Compared to other people, do you consider yourself allergic or unusually sensitive to everyday chemicals like those in household cleaning products, paints, perfumes, detergents, insect spray and things like that?”

To ascertain asthma, the survey asked “Has a doctor or health care professional ever told you that you have asthma or an asthma-like condition?” and then further asked to specify whether asthma or an asthma-like condition. The term “asthmatic” will be used herein to encompass individuals with either asthma or an asthma-like condition or both.

To ascertain fragrance sensitivity, the survey investigated health effects associated with exposures to fragranced consumer products. A “fragranced consumer product,” or “fragranced product” for brevity, is a chemically formulated product with the addition of a fragrance or scent.^6^ An individual was considered to characterize fragrance sensitivity if they experienced one or more types of health problems from one or more types of fragranced products and exposure contexts.

Fragranced product types were categorized as follows: air fresheners and deodorizers, personal care products, cleaning supplies, laundry products, household products, fragrance, and other. Health effects were categorized as follows: migraine headaches; asthma attacks; neurological problems; respiratory problems; skin problems; cognitive problems; mucosal symptoms; immune system problems; gastrointestinal problems; cardiovascular problems; musculoskeletal problems; and other. (Additional details on specific product types and health effects within each category, along with response data, are provided in the file “Survey Data” as Supplemental Digital Content.)

Specific exposure contexts were air fresheners or deodorizers used in public restrooms and other environments, scented laundry products coming from a dryer vent, being in a room after it was cleaned with scented cleaning products, being near someone wearing a fragranced product, entering a business with the scent of fragranced products, fragranced soap used in public restrooms, and ability to access environments that used fragranced products. The survey also investigated effects of fragrance exposure in the workplace, access to public places that used fragranced products, and preferences for fragrance-free environments and policies. Data on fragranced product exposures and effects were derived from a survey of the general population,^6^ while the present study focuses specifically on effects on the subpopulations of individuals with MCS or chemical sensitivity.

## RESULTS

A national prevalence of 12.8% medically diagnosed MCS, 25.9% self-reported chemical sensitivity, and 27.5% either or both, was assessed by the survey (See Table 1). Compared with previous studies,^4,5^ the prevalence of diagnosed MCS has increased over three times (2.5%, 3.9% to 12.8%) and self-reported chemical sensitivity has increased over two times (11.1%, 11.6% to 25.9%) in a little over 10 years.

In addition, 71.0% of those with MCS are asthmatic: diagnosed with asthma (40.0%), an asthma-like condition (34.5%), or both. Also, 59.2% with chemical sensitivity are asthmatic: diagnosed with asthma (35.0%), an asthma-like condition (26.2%), or both (See Table 1). Compared with previous studies,^4,5^ the co-occurrence of asthma with diagnosed MCS (42.3%, 39.0% vs 40.0%) and with chemical sensitivity (34.2%, 34.9% vs 35.0%) is relatively similar.

Fragranced consumer products were found to trigger a range of adverse health and societal effects. When exposed to fragranced consumer products, 86.2% of those with MCS experience one or more types of health problems, including respiratory difficulties (50.3%), migraine headaches (46.9%), mucosal symptoms (46.9%), skin problems (37.9%), and asthma attacks (31.7%). Similarly, 81.2% of those with chemical sensitivity report one or more types of health problems when exposed to fragranced products (see Tables 1 and 2).

Specific exposures triggering health problems include air fresheners and deodorizers (67.6%), scented laundry products coming from a dryer vent (57.9%), being in a room recently cleaned with scented products (67.6%), being near someone wearing a fragranced product (65.5%), and in general fragranced consumer products (73.1%) (see Table 3, and the file “Survey Data” as Supplemental Digital Content).

For 76.0% of people with MCS, the severity of these health problems was potentially disabling according to the criterion of the Americans with Disabilities Act Amendments Act of 2008 (ADAAA), asked by the question: “Do any of these health problems substantially limit one or more major life activities, such as seeing, hearing, eating, sleeping, walking, standing, lifting, bending, speaking, breathing, learning, reading, concentrating, thinking, communicating, or working, for you personally?”^16^ (See Table 4).

Fragranced products also restrict access in society: 58.6% of individuals with MCS are unable to use public restrooms that have an air freshener, deodorizer, or scented product; 55.2% are unable to wash their hands in a public place if the soap is fragranced; 63.4% enter a business but then want to leave as quickly as possible due to a fragranced product; and 70.3% have been prevented from going someplace because of the presence of a fragranced product that would make them sick (See Table 4).

Significantly, 60.7% of those with MCS lost workdays or a job in the past year due to illness from fragranced product exposure in the workplace. Further, 71% of those with MCS would support a fragrance-free policy in the workplace, and 82.1% would prefer that health care facilities and professionals were fragrance-free (See Table 4).

Demographic proportions of diagnosed MCS are 57.9% male and 42.1% female, compared with the general population of 46.2% male and 53.8% female. Thus, diagnosed MCS has a male bias (+11.7%). Previous national prevalence studies in the US found instead a slight female bias. Relative to gender and age, the highest bias (percentage MCS greater than general population) is male 25 to 34 (+12.7%) (See Table 5).

## DISCUSSION

Results of this study provide evidence that MCS is widespread and increasing in the US population: an estimated 25.6 million adults are diagnosed with MCS, and an estimated 51.8 million adults report chemical sensitivity.^17^ Using the same criteria to assess MCS and chemical sensitivity as prior US national prevalence studies, this represents an increase of 300% in diagnosed MCS and 200% in self-reported chemical sensitivity in a little more than 10 years.

Among individuals diagnosed with MCS, 71.0% report being diagnosed also with asthma or an asthma-like condition. Thus, individuals with MCS are proportionally more likely to be asthmatic than individuals without MCS (prevalence odds ratio 9.6; 95% confidence interval 6.5 to 14.2).

In addition, among individuals with MCS, 86.2% report adverse health effects from exposure to fragranced consumer products. Thus, individuals with MCS are proportionally more likely to be fragrance sensitive than individuals without MCS (prevalence odds ratio 16.8; 95% confidence interval 10.3 to 27.5).

As a consequence, individuals with MCS are prevented from accessing restrooms, businesses, workplaces, and public places due to risk of adverse health effects—some potentially disabling—from fragranced consumer products. Notably, exposure to fragranced consumer products is associated with lost workdays or a job, in the past year, for 11.0% of the adult population with MCS or chemical sensitivity, representing an estimated 22 million Americans. While researchers continue to investigate which chemicals or mixtures of chemicals in fragranced consumer products could be associated with adverse effects,^18^ a practical step in the meantime would be to reduce exposure to the products. For instance, 71.0% of those with MCS would support fragrance-free policies in the workplace, and 82.1% would prefer fragrance-free health care facilities and professionals, as would a majority of the US general population.^6^

Limitations of the study include the following: (a) data were based on self-reports, although a standard and accepted method for epidemiological research, and consistent with prior prevalence studies of MCS; (b) only adults (ages 18 to 65) were surveyed; (c) all possible fragranced products and health effects were not included, although the low percentages for responses in the “other” category indicates the survey captured the primary products and effects; and (d) MCS and chemical sensitivity lack standard diagnostic criteria, although the survey replicated questions from prior large-scale USA prevalence studies to promote comparability.

## CONCLUSION

The prevalence of MCS has increased across the American population, and it frequently co-occurs with asthma and fragrance sensitivity. Moreover, fragranced consumer products, such as air fresheners and scented cleaning products, trigger significant adverse health and societal effects among individuals with MCS. Reducing exposure to fragranced products, such as through fragrance-free policies, would be an important practical step to reduce the adverse effects.

## Supplementary Material

Supplemental Digital Content

## Supplementary Material

Supplemental Digital Content

## Figures and Tables

**TABLE 1:** Prevalence and Co-Occurrence of MCS and Chemical Sensitivity With Asthma and Fragrance Sensitivity

	Gen Pop	MCS Diag	ChemSens	MCS/ChemSens
Total (*N*)	1,137	145	294	313
(% relative to general population)	100.0%	12.8%	25.9%	27.5%

	*N*	*N*	*N*	*N*
	% of Column Total	% of Column Total	% of Column Total	% of Column Total
MCS diagnosed	145	145	126	145
	12.8%	100.0%	42.9%	46.3%
Chemically sensitive	294	126	294	294
	25.9%	86.9%	100.0%	93.9%
MCS diagnosed or chemically sensitive (or both)	313	145	294	313
	27.5%	100.0%	100.0%	100.0%
Asthma diagnosed	173	58	103	105
	15.2%	40.0%	35.0%	33.5%
Asthma-like condition diagnosed	142	50	77	80
	12.5%	34.5%	26.2%	25.6%
Asthmatic (asthma or asthma-like condition or both)	305	103	174	179
	26.8%	71.0%	59.2%	57.2%
Fragrance sensitive	394	125	238	247
	34.7%	86.2%	81.0%	78.9%

ChemSens, self-reported chemical sensitivity; Gen Pop, general population (including subpopulations of MCS and ChemSens); MCS Diag, medically diagnosed with MCS; MCS/ChemSens, medically diagnosed with MCS, or self-reported chemical sensitivity, or both.

**TABLE 2:** Health Problems (Frequency and Type) Reported from Exposure to Fragranced Consumer Products

	Gen Pop	MCS Diag	ChemSens	MCS/ChemSens
Total (*N*)	1,137	145	294	313
(% relative to general population)	100.0%	12.8%	25.9%	27.5%

	*N*	*N*	*N*	*N*
	% of Column Total	% of Column Total	% of Column Total	% of Column Total
Total fragrance sensitive (*N*) (reporting one or more health problems)(% relative to Subpopulation)	39434.7%	12586.2%	23881.0%	24778.9%
Type of health problem				
^*^ Migraine headaches	179	68	124	128
	15.7%	46.9%	42.2%	40.9%
^*^ Asthma attacks	91	46	75	75
	8.0%	31.7%	25.5%	24.0%
^*^ Neurological problems (eg, dizziness, seizures, head pain, fainting, loss of coordination)	827.2%	3826.2%	6221.1%	6320.1%
^*^ Respiratory problems (eg, difficulty breathing, coughing, shortness of breath)	21118.6%	7350.3%	14750.0%	14847.3%
^*^ Skin problems (eg, rashes, hives, red skin, tingling skin, dermatitis)	12110.6%	5537.9%	8428.6%	8828.1%
^*^ Cognitive problems (eg, difficulties thinking, concentrating, or remembering)	665.8%	3524.1%	5619.0%	5718.2%
^*^ Mucosal symptoms (eg, watery or red eyes, nasal congestion, sneezing)	18416.2%	6846.9%	12040.8%	12439.6%
^*^ Immune system problems (eg, swollen lymph glands, fever, fatigue)	454.0%	3121.4%	3913.3%	3912.5%
^*^ Gastrointestinal problems (eg, nausea, bloating, cramping, diarrhea)	635.5%	3222.1%	5318.0%	5316.9%
^*^ Cardiovascular problems (eg, fast or irregular heartbeat, jitteriness, chest discomfort)	504.4%	2819.3%	3712.6%	3812.1%
^*^ Musculoskeletal problems (eg, muscle or joint pain, cramps, weakness)	433.8%	2819.3%	3511.9%	3611.5%
^*^ Other	19	2	6	6
	1.7%	1.4%	2.0%	1.9%

**TABLE 3:** Health Problems (Frequency and Type) Reported from Exposure to Fragranced Consumer Products

	Gen Pop	MCS Diag	ChemSens	MCS/ChemSens
	*N*	*N*	*N*	*N*
	% of Column Total	% of Column Total	% of Column Total	% of Column Total
Total	1,137	145	294	313
	100.0%	100.0%	100.0%	100.0%
Fragrance sensitive	394	125	238	247
	34.7%	86.2%	81.0%	78.9%
Health problems from exposure to				
Air fresheners or deodorizers	232	98	162	168
	20.4%	67.6%	55.1%	53.7%
Scented laundry products from a dryer vent	142	84	107	112
	12.5%	57.9%	36.4%	35.8%
Room cleaned with scented products	224	98	166	171
	19.7%	67.6%	56.5%	54.6%
Someone wearing a fragranced product	268	95	178	183
	23.6%	65.5%	60.5%	58.5%
Any type of fragranced consumer product	253	106	192	196
	22.3%	73.1%	65.3%	62.6%

**TABLE 4:** Societal Effects of Fragranced Consumer Products for Individuals with MCS

	Gen Pop	MCS Diag	ChemSens	MCS/ChemSens
	*N*	*N*	*N*	*N*
	% of Column Total	% of Column Total	% of Column Total	% of Column Total
Total	1,137	145	294	313
	100.0%	100.0%	100.0%	100.0%
Fragrance sensitive	394	125	238	247
	34.7%	86.2%	81.0%	78.9%
Disabling health effects from fragranced consumer products	195	95	160	164
	49.5%	76.0%	67.2%	66.4%
Unable to use restrooms in public place because of air freshener, deodorizer, or scented product	19917.5%	8558.6%	13244.9%	13844.1%
Unable to wash hands because of fragranced soap	160	80	118	122
	14.1%	55.2%	40.1%	39.0%
Want to leave a business quickly because of fragranced product	22920.1%	9263.4%	16054.4%	16452.4%
Prevented from going someplace because of fragranced product	25822.7%	10270.3%	16857.1%	17957.2%
Lost workdays or job in past year due to fragranced product exposure in workplace	17215.1%	8860.7%	11940.5%	12539.9%
Supportive of fragrance-free policy in the workplace	604	103	212	221
	53.1%	71.0%	72.1%	70.6%
Prefer fragrance-free health care facilities and professionals	623	119	236	248
	54.8%	82.1%	80.3%	79.2%

**TABLE 5:** Demographic Information

	Gen Pop	MCS Diag	ChemSens	ChemSens/MCS
	*N*	*N*	*N*	*N*
	% of Column Total	% of Column Total	% of Column Total	% of Column Total
Total	1,137	145	294	313
	100.0%	100.0%	100.0%	100.0%
Male/Female				
All males	525	84	133	145
	46.2%	57.9%	45.2%	46.3%
All females	612	61	161	168
	53.8%	42.1%	54.8%	53.7%
Gender vs age				
Male 18–24	47	7	10	12
	4.1%	4.8%	3.4%	3.8%
Male 25–34	130	35	42	47
	11.4%	24.1%	14.3%	15.0%
Male 35–44	136	30	44	48
	12.0%	20.7%	15.0%	15.3%
Male 45–54	108	4	20	20
	9.5%	2.8%	6.8%	6.4%
Male 55–65	104	8	17	18
	9.1%	5.5%	5.8%	5.8%
Female 18–24	78	8	19	21
	6.9%	5.5%	6.5%	6.7%
Female 25–34	135	16	34	35
	11.9%	11.0%	11.6%	11.2%
Female 35–44	155	16	45	47
	13.6%	11.0%	15.3%	15.0%
Female 45–54	144	13	41	42
	12.7%	9.0%	13.9%	13.4%
Female 55–65	100	8	22	23
	8.8%	5.5%	7.5%	7.3%
