# Supplementary material for: National Prevalence and Effects of Multiple Chemical Sensitivities
Source: J Occup Environ Med. 2018 Jan 12;60(3):e152–6. doi: 10.1097/JOM.0000000000001272 (PMC5865484; doi:10.1097/JOM.0000000000001272)
Supplement: Supplemental Digital Content [file joem-60-e152-s002.pdf]

Table 1

What is your gender?

	Gen Pop	MCS Diag	ChemSens	ChemSens/MCS
Total	1137	145	294	313
	100.00%	100.00%	100.00%	100.00%
Male	525	84	133	145
	46.20%	57.90%	45.20%	46.30%
Female	612	61	161	168
	53.80%	42.10%	54.80%	53.70%
Other	-	-	-	-
	-	-	-	-

Table 2

What is your age?

	Gen Pop	MCS Diag	ChemSens	ChemSens/MCS
Total	1137	145	294	313
	100.00%	100.00%	100.00%	100.00%
18-24 (21)	125	15	29	33
	11.00%	10.30%	9.90%	10.50%
25-34 (29.5)	265	51	76	82
	23.30%	35.20%	25.90%	26.20%
35-44 (39.5)	291	46	89	95
	25.60%	31.70%	30.30%	30.40%
45-54 (49.5)	252	17	61	62
	22.20%	11.70%	20.70%	19.80%
55-65 (60)	204	16	39	41
	17.90%	11.00%	13.30%	13.10%
65 (65)	-	-	-	-

Table 3

USA Region

	Gen Pop	MCS Diag	ChemSens	ChemSens/MCS
Total	1137	145	294	313
	100.00%	100.00%	100.00%	100.00%
Northeast	207	24	50	53
	18.20%	16.60%	17.00%	16.90%
Midwest	246	26	57	59
	21.60%	17.90%	19.40%	18.80%
South	422	51	107	115
	37.10%	35.20%	36.40%	36.70%
West	262	44	80	86
	23.00%	30.30%	27.20%	27.50%
SUM	1137	145	294	313
	100.00%	100.00%	100.00%	100.00%

Table 4

QA. Do you experience any health problems when exposed to air fresheners or deodorizers?

	Gen Pop	MCS Diag	ChemSens	ChemSens/MCS
Total	1137	145	294	313
	100.00%	100.00%	100.00%	100.00%
Yes	232	98	162	168
	20.40%	67.60%	55.10%	53.70%
No	791	36	88	98
	69.60%	24.80%	29.90%	31.30%
Don't know/not sure	109	11	43	46
	9.60%	7.60%	14.60%	14.70%
Decline to answer	5	-	1	1
	0.40%	-	0.30%	0.30%
SUM	1137	145	294	313
	100.00%	100.00%	100.00%	100.00%

Table 5

Which of the following health problems do you experience?

Base: Respondents who experienced below health problems when exposed to air fresheners or deodorizers

	Gen Pop	MCS Diag	ChemSens	ChemSens/MCS
Total	232	98	162	168
	100.00%	100.00%	100.00%	100.00%
Migraine headaches	82	36	65	65
	35.30%	36.70%	40.10%	38.70%
Asthma attacks	53	27	44	44
	22.80%	27.60%	27.20%	26.20%
Neurological problems (e.g., dizziness, seizures,	36	20	29	29
head pain, fainting, loss of coordination)	15.50%	20.40%	17.90%	17.30%
Respiratory problems (e.g., difficulty breathing,	108	46	82	83
coughing, shortness of breath)	46.60%	46.90%	50.60%	49.40%
Skin problems (e.g., rashes, hives, red skin,	65	33	49	52
tingling skin, dermatitis)	28.00%	33.70%	30.20%	31.00%
Cognitive problems (e.g., difficulties thinking,	31	16	26	26
concentrating, or remembering)	13.40%	16.30%	16.00%	15.50%
nasal	86	37	63	66
congestion, sneezing)	37.10%	37.80%	38.90%	39.30%
Immune system problems (e.g., swollen lymph	21	13	17	17
glands, fever, fatigue)	9.10%	13.30%	10.50%	10.10%
bloating,	31	18	26	26
cramping, diarrhea)	13.40%	18.40%	16.00%	15.50%
Cardiovascular problems (e.g., fast or irregular	30	18	22	22
heartbeat, jitteriness, chest discomfort)	12.90%	18.40%	13.60%	13.10%
Musculoskeletal problems (e.g., muscle or joint	27	18	22	22
pain, cramps, weakness)	11.60%	18.40%	13.60%	13.10%
Other	8	2	3	3
	3.40%	2.00%	1.90%	1.80%
SUM	578	284	448	455
	249.10%	289.80%	276.50%	270.80%

Table 6

QB. Do you experience any health problems from the scent of laundry products coming from a dryer vent?

	Gen Pop	MCS Diag	ChemSens	ChemSens/MCS
Total	1137	145	294	313
	100.00%	100.00%	100.00%	100.00%
Yes	142	84	107	112
	12.50%	57.90%	36.40%	35.80%
No	906	52	143	157
	79.70%	35.90%	48.60%	50.20%
Don't know/not sure	88	9	44	44
	7.70%	6.20%	15.00%	14.10%
Decline to answer	1	-	-	-
	0.10%	-	-	-
SUM	1137	145	294	313
	100.00%	100.00%	100.00%	100.00%

Table 7

Which of the following health problems do you experience?

Base: Respondents who experienced below health problems from the scent of laundry products coming from a dryer vent

	Gen Pop	MCS Diag	ChemSens	ChemSens/MCS
Total	142	84	107	112
	100.00%	100.00%	100.00%	100.00%
Migraine headaches	37	23	28	29
	26.10%	27.40%	26.20%	25.90%
Asthma attacks	28	21	25	25
	19.70%	25.00%	23.40%	22.30%
Neurological problems (e.g., dizziness, seizures,	24	19	21	22
head pain, fainting, loss of coordination)	16.90%	22.60%	19.60%	19.60%
Respiratory problems (e.g., difficulty breathing,	46	31	39	40
coughing, shortness of breath)	32.40%	36.90%	36.40%	35.70%
Skin problems (e.g., rashes, hives, red skin,	41	22	26	26
tingling skin, dermatitis)	28.90%	26.20%	24.30%	23.20%
Cognitive problems (e.g., difficulties thinking,	15	10	13	13
concentrating, or remembering)	10.60%	11.90%	12.10%	11.60%
nasal	48	29	42	43
congestion, sneezing)	33.80%	34.50%	39.30%	38.40%
Immune system problems (e.g., swollen lymph	19	14	17	17
glands, fever, fatigue)	13.40%	16.70%	15.90%	15.20%
bloating,	29	18	26	26
cramping, diarrhea)	20.40%	21.40%	24.30%	23.20%
Cardiovascular problems (e.g., fast or irregular	15	11	13	13
heartbeat, jitteriness, chest discomfort)	10.60%	13.10%	12.10%	11.60%
Musculoskeletal problems (e.g., muscle or joint	23	19	21	22
pain, cramps, weakness)	16.20%	22.60%	19.60%	19.60%
Other	4	1	2	2
	2.80%	1.20%	1.90%	1.80%
SUM	329	218	273	278
	231.70%	259.50%	255.10%	248.20%

Table 8

QC. Do you experience any health problems from being in a room after it has been cleaned with scented products?

	Gen Pop	MCS Diag	ChemSens	ChemSens/MCS
Total	1137	145	294	313
	100.00%	100.00%	100.00%	100.00%
Yes	224	98	166	171
	19.70%	67.60%	56.50%	54.60%
No	839	42	105	118
	73.80%	29.00%	35.70%	37.70%
Don't know/not sure	73	5	23	24
	6.40%	3.40%	7.80%	7.70%
Decline to answer	1	-	-	-
	0.10%	-	-	-
SUM	1137	145	294	313
	100.00%	100.00%	100.00%	100.00%

Table 9

Which of the following health problems do you experience?

Base: Respondents who experienced below health problems from being in a room after it has been cleaned with scented products

	Gen Pop	MCS Diag	ChemSens	ChemSens/MCS
Total	224	98	166	171
	100.00%	100.00%	100.00%	100.00%
Migraine headaches	75	33	59	60
	33.50%	33.70%	35.50%	35.10%
Asthma attacks	46	23	38	38
	20.50%	23.50%	22.90%	22.20%
Neurological problems (e.g., dizziness, seizures,	47	23	39	39
head pain, fainting, loss of coordination)	21.00%	23.50%	23.50%	22.80%
Respiratory problems (e.g., difficulty breathing,	109	40	81	82
coughing, shortness of breath)	48.70%	40.80%	48.80%	48.00%
Skin problems (e.g., rashes, hives, red skin,	45	27	33	35
tingling skin, dermatitis)	20.10%	27.60%	19.90%	20.50%
Cognitive problems (e.g., difficulties thinking,	31	20	28	28
concentrating, or remembering)	13.80%	20.40%	16.90%	16.40%
nasal	83	33	60	63
congestion, sneezing)	37.10%	33.70%	36.10%	36.80%
Immune system problems (e.g., swollen lymph	23	17	20	20
glands, fever, fatigue)	10.30%	17.30%	12.00%	11.70%
bloating,	32	16	28	28
cramping, diarrhea)	14.30%	16.30%	16.90%	16.40%
Cardiovascular problems (e.g., fast or irregular	26	17	22	23
heartbeat, jitteriness, chest discomfort)	11.60%	17.30%	13.30%	13.50%
Musculoskeletal problems (e.g., muscle or joint	23	16	19	19
pain, cramps, weakness)	10.30%	16.30%	11.40%	11.10%
Other	4	1	2	2
	1.80%	1.00%	1.20%	1.20%
SUM	544	266	429	437
	242.90%	271.40%	258.40%	255.60%

Table 10

QD. Do you experience any health problems from being near someone who is wearing a fragranced product?

	Gen Pop	MCS Diag	ChemSens	ChemSens/MCS
Total	1137	145	294	313
	100.00%	100.00%	100.00%	100.00%
Yes	268	95	178	183
	23.60%	65.50%	60.50%	58.50%
No	799	44	92	104
	70.30%	30.30%	31.30%	33.20%
Don't know/not sure	68	6	24	26
	6.00%	4.10%	8.20%	8.30%
Decline to answer	2	-	-	-
	0.20%	-	-	-
SUM	1137	145	294	313
	100.00%	100.00%	100.00%	100.00%

Table 11

Which of the following health problems do you experience?

Base: Respondents who experienced below health problems from being near someone who is wearing a fragranced product

	Gen Pop	MCS Diag	ChemSens	ChemSens/MCS
Total	268	95	178	183
	100.00%	100.00%	100.00%	100.00%
Migraine headaches	96	38	69	70
	35.80%	40.00%	38.80%	38.30%
Asthma attacks	44	23	36	36
	16.40%	24.20%	20.20%	19.70%
Neurological problems (e.g., dizziness, seizures,	41	19	34	34
head pain, fainting, loss of coordination)	15.30%	20.00%	19.10%	18.60%
Respiratory problems (e.g., difficulty breathing,	118	43	83	84
coughing, shortness of breath)	44.00%	45.30%	46.60%	45.90%
Skin problems (e.g., rashes, hives, red skin,	39	24	31	33
tingling skin, dermatitis)	14.60%	25.30%	17.40%	18.00%
Cognitive problems (e.g., difficulties thinking,	30	17	27	28
concentrating, or remembering)	11.20%	17.90%	15.20%	15.30%
nasal	98	34	60	63
congestion, sneezing)	36.60%	35.80%	33.70%	34.40%
Immune system problems (e.g., swollen lymph	19	16	19	19
glands, fever, fatigue)	7.10%	16.80%	10.70%	10.40%
bloating,	31	17	29	29
cramping, diarrhea)	11.60%	17.90%	16.30%	15.80%
Cardiovascular problems (e.g., fast or irregular	20	14	18	18
heartbeat, jitteriness, chest discomfort)	7.50%	14.70%	10.10%	9.80%
Musculoskeletal problems (e.g., muscle or joint	17	13	16	16
pain, cramps, weakness)	6.30%	13.70%	9.00%	8.70%
Other	7	1	3	3
	2.60%	1.10%	1.70%	1.60%
SUM	560	259	425	433
	209.00%	272.60%	238.80%	236.60%

Table 12

QE. In general, do you experience any health problems from exposure to any type of fragranced product?

	Gen Pop	MCS Diag	ChemSens	ChemSens/MCS
Total	1137	145	294	313
	100.00%	100.00%	100.00%	100.00%
Yes	253	106	192	196
	22.30%	73.10%	65.30%	62.60%
No	796	30	77	88
	70.00%	20.70%	26.20%	28.10%
Don't know/not sure	87	9	25	29
	7.70%	6.20%	8.50%	9.30%
Decline to answer	1	-	-	-
	0.10%	-	-	-
SUM	1137	145	294	313
	100.00%	100.00%	100.00%	100.00%

Table 13

Which of the following health problems do you experience?

Base: Respondents who experienced below health problems from exposure to any type of fragranced product

	Gen Pop	MCS Diag	ChemSens	ChemSens/MCS
Total	253	106	192	196
	100.00%	100.00%	100.00%	100.00%
Migraine headaches	98	38	76	78
	38.70%	35.80%	39.60%	39.80%
Asthma attacks	55	30	48	48
	21.70%	28.30%	25.00%	24.50%
Neurological problems (e.g., dizziness, seizures,	42	23	38	39
head pain, fainting, loss of coordination)	16.60%	21.70%	19.80%	19.90%
Respiratory problems (e.g., difficulty breathing,	119	45	94	95
coughing, shortness of breath)	47.00%	42.50%	49.00%	48.50%
Skin problems (e.g., rashes, hives, red skin,	58	32	49	50
tingling skin, dermatitis)	22.90%	30.20%	25.50%	25.50%
Cognitive problems (e.g., difficulties thinking,	31	23	29	29
concentrating, or remembering)	12.30%	21.70%	15.10%	14.80%
nasal	102	41	76	78
congestion, sneezing)	40.30%	38.70%	39.60%	39.80%
Immune system problems (e.g., swollen lymph	24	21	24	24
glands, fever, fatigue)	9.50%	19.80%	12.50%	12.20%
bloating,	33	18	30	30
cramping, diarrhea)	13.00%	17.00%	15.60%	15.30%
Cardiovascular problems (e.g., fast or irregular	18	12	16	16
heartbeat, jitteriness, chest discomfort)	7.10%	11.30%	8.30%	8.20%
Musculoskeletal problems (e.g., muscle or joint	20	15	18	18
pain, cramps, weakness)	7.90%	14.20%	9.40%	9.20%
Other	3	1	2	2
	1.20%	0.90%	1.00%	1.00%
SUM	603	299	500	507
	238.30%	282.10%	260.40%	258.70%

Table 14

Do any of these health problems substantially limit one or more major life activities, such as seeing, hearing, eating, sleeping, walking, standing, lifting, bending, speaking, breathing, learning, reading, concentrating, thinking, communicating, or working, for you personally? (ADA)

	Gen Pop	MCS Diag	ChemSens	ChemSens/MCS
Total	394	125	238	247
	100.00%	100.00%	100.00%	100.00%
Yes	195	95	160	164
	49.50%	76.00%	67.20%	66.40%
No	175	27	69	72
	44.40%	21.60%	29.00%	29.10%
Don't know/not sure	22	2	9	10
	5.60%	1.60%	3.80%	4.00%
Decline to answer	2	1	-	1
	0.50%	0.80%	-	0.40%
SUM	394	125	238	247
	100.00%	100.00%	100.00%	100.00%

Table 15

Compared to other people, do you consider yourself allergic or unusually sensitive to everyday chemicals like those in household cleaning products, paints, perfumes, detergents, insect spray and things like that?

	Gen Pop	MCS Diag	ChemSens	ChemSens/MCS
Total	1137	145	294	313
	100.00%	100.00%	100.00%	100.00%
Yes	294	126	294	294
	25.90%	86.90%	100.00%	93.90%
No	773	17	-	17
	68.00%	11.70%	-	5.40%
Don't know/not sure	68	2	-	2
	6.00%	1.40%	-	0.60%
Decline to answer	2	-	-	-
	0.20%	-	-	-
SUM	1137	145	294	313
	100.00%	100.00%	100.00%	100.00%

Table 16

Has a doctor or health care professional ever told you that you have multiple chemical sensitivities?

	Gen Pop	MCS Diag	ChemSens	ChemSens/MCS
Total	1137	145	294	313
	100.00%	100.00%	100.00%	100.00%
Yes	145	145	126	145
	12.80%	100.00%	42.90%	46.30%
No	950	-	151	151
	83.60%	-	51.40%	48.20%
Don't know/not sure	40	-	17	17
	3.50%	-	5.80%	5.40%
Decline to answer	2	-	-	-
	0.20%	-	-	-
SUM	1137	145	294	313
	100.00%	100.00%	100.00%	100.00%

Table 17

Has a doctor or health care professional ever told you that you have asthma or an asthma-like condition?

	Gen Pop	MCS Diag	ChemSens	ChemSens/MCS
Total	1137	145	294	313
	100.00%	100.00%	100.00%	100.00%
Yes - asthma	173	58	103	105
	15.20%	40.00%	35.00%	33.50%
Yes - asthma-like condition	142	50	77	80
	12.50%	34.50%	26.20%	25.60%
No	811	39	114	126
	71.30%	26.90%	38.80%	40.30%
Don't know/not sure	19	3	6	8
	1.70%	2.10%	2.00%	2.60%
Decline to answer	2	-	-	-
	0.20%	-	-	-
SUM	1147	150	300	319
	100.90%	103.40%	102.00%	101.90%

Table 18

People Who Answer “Yes” to "Asthma" or "Asthma-Like Condition"

	Gen Pop	MCS Diag	ChemSens	ChemSens/MCS
Total	1137	145	294	313
	100.00%	100.00%	100.00%	100.00%
Yes	305	103	174	179
	26.80%	71.00%	59.20%	57.20%

Table 19

People Who Answer “Yes” To One Or More Of These Questions: QA/QB/QC/QD/QE (fragrance sensitive group).

	Gen Pop	MCS Diag	ChemSens	ChemSens/MCS
Total	1137	145	294	313
	100.00%	100.00%	100.00%	100.00%
Yes	394	125	238	247
	34.70%	86.20%	81.00%	78.90%

Table 20

Which of the following health problems do you experience?

Base: Yes to QA/QB/QC/QD/QE (fragrance sensitive group)

	Gen Pop	MCS Diag	ChemSens	ChemSens/MCS
Total	1137	145	294	313
	100.00%	100.00%	100.00%	100.00%
Migraine headaches	179	68	124	128
	15.70%	46.90%	42.20%	40.90%
Asthma attacks	91	46	75	75
	8.00%	31.70%	25.50%	24.00%
Neurological problems (e.g., dizziness, seizures,	82	38	62	63
head pain, fainting, loss of coordination)	7.20%	26.20%	21.10%	20.10%
Respiratory problems (e.g., difficulty breathing,	211	73	147	148
coughing, shortness of breath)	18.60%	50.30%	50.00%	47.30%
Skin problems (e.g., rashes, hives, red skin,	121	55	84	88
tingling skin, dermatitis)	10.60%	37.90%	28.60%	28.10%
Cognitive problems (e.g., difficulties thinking,	66	35	56	57
concentrating, or remembering)	5.80%	24.10%	19.00%	18.20%
nasal	184	68	120	124
congestion, sneezing)	16.20%	46.90%	40.80%	39.60%
Immune system problems (e.g., swollen lymph	45	31	39	39
glands, fever, fatigue)	4.00%	21.40%	13.30%	12.50%
bloating,	63	32	53	53
cramping, diarrhea)	5.50%	22.10%	18.00%	16.90%
Cardiovascular problems (e.g., fast or irregular	50	28	37	38
heartbeat, jitteriness, chest discomfort)	4.40%	19.30%	12.60%	12.10%
Musculoskeletal problems (e.g., muscle or joint	43	28	35	36
pain, cramps, weakness)	3.80%	19.30%	11.90%	11.50%
Other	19	2	6	6
	1.70%	1.40%	2.00%	1.90%

Table 21

Would you be supportive of a fragrance-free policy in the workplace?

	Gen Pop	MCS Diag	ChemSens	ChemSens/MCS
Total	1137	145	294	313
	100.00%	100.00%	100.00%	100.00%
Yes	604	103	212	221
	53.10%	71.00%	72.10%	70.60%
No	224	25	35	40
	19.70%	17.20%	11.90%	12.80%
Neutral/not sure	304	16	47	51
	26.70%	11.00%	16.00%	16.30%
Decline to answer	5	1	-	1
	0.40%	0.70%	-	0.30%
SUM	1137	145	294	313
	100.00%	100.00%	100.00%	100.00%

Table 22

Would you prefer that health care facilities and health care professionals be fragrance-free?

	Gen Pop	MCS Diag	ChemSens	ChemSens/MCS
Total	1137	145	294	313
	100.00%	100.00%	100.00%	100.00%
Yes	623	119	236	248
	54.80%	82.10%	80.30%	79.20%
No	255	15	33	37
	22.40%	10.30%	11.20%	11.80%
Neutral/not sure	254	11	25	28
	22.30%	7.60%	8.50%	8.90%
Decline to answer	5	-	-	-
	0.40%	-	-	-
SUM	1137	145	294	313
	100.00%	100.00%	100.00%	100.00%

Table 23

Have you ever been prevented from going to some place because you would be exposed to a fragrance product that would make you sick?

	Gen Pop	MCS Diag	ChemSens	ChemSens/MCS
Total	1137	145	294	313
	100.00%	100.00%	100.00%	100.00%
Yes	258	102	168	179
	22.70%	70.30%	57.10%	57.20%
No	819	36	106	113
	72.00%	24.80%	36.10%	36.10%
Don't know/not sure	58	6	20	20
	5.10%	4.10%	6.80%	6.40%
Decline to answer	2	1	-	1
	0.20%	0.70%	-	0.30%
SUM	1137	145	294	313
	100.00%	100.00%	100.00%	100.00%

Table 24

Has any exposure to fragranced products in your work environment caused you to become sick, lose work days, or lose a job?

	Gen Pop	MCS Diag	ChemSens	ChemSens/MCS
Total	1137	145	294	313
	100.00%	100.00%	100.00%	100.00%
Yes	172	88	119	125
	15.10%	60.70%	40.50%	39.90%
No	910	53	159	170
	80.00%	36.60%	54.10%	54.30%
Don't know/not sure	54	4	16	18
	4.70%	2.80%	5.40%	5.80%
Decline to answer	1	-	-	-
	0.10%	-	-	-
SUM	1137	145	294	313
	100.00%	100.00%	100.00%	100.00%

Table 25

Have you ever been unable or reluctant to use the toilets in a public place, because of the presence of an air freshener, deodorizer, or scented product?

	Gen Pop	MCS Diag	ChemSens	ChemSens/MCS
Total	1137	145	294	313
	100.00%	100.00%	100.00%	100.00%
Yes	199	85	132	138
	17.50%	58.60%	44.90%	44.10%
No	897	55	146	159
	78.90%	37.90%	49.70%	50.80%
Neutral/not sure	40	5	16	16
	3.50%	3.40%	5.40%	5.10%
Decline to answer	1	-	-	-
	0.10%	-	-	-
SUM	1137	145	294	313
	100.00%	100.00%	100.00%	100.00%

Table 26

If you enter a business, and you smell air fresheners or some fragranced product, do you want to leave as quickly as possible?

	Gen Pop	MCS Diag	ChemSens	ChemSens/MCS
Total	1137	145	294	313
	100.00%	100.00%	100.00%	100.00%
Yes	229	92	160	164
	20.10%	63.40%	54.40%	52.40%
No	787	38	91	103
	69.20%	26.20%	31.00%	32.90%
Neutral/not sure	120	15	43	46
	10.60%	10.30%	14.60%	14.70%
Decline to answer	1	-	-	-
	0.10%	-	-	-
SUM	1137	145	294	313
	100.00%	100.00%	100.00%	100.00%

Table 27

Have you ever been unable or reluctant to wash your hands with soap in a public place, because you know or suspect that the soap is fragranced?

	Gen Pop	MCS Diag	ChemSens	ChemSens/MCS
Total	1137	145	294	313
	100.00%	100.00%	100.00%	100.00%
Yes	160	80	118	122
	14.10%	55.20%	40.10%	39.00%
No	924	53	159	170
	81.30%	36.60%	54.10%	54.30%
Neutral/not sure	50	12	17	21
	4.40%	8.30%	5.80%	6.70%
Decline to answer	3	-	-	-
	0.30%	-	-	-
SUM	1137	145	294	313
	100.00%	100.00%	100.00%	100.00%

Table 28

Demographic information

	Gen Pop	MCS Diag	ChemSens	ChemSens/MCS
Total	1137	145	294	313
	100.00%	100.00%	100.00%	100.00%
**Male/Female**				
All Males	525	84	133	145
	46.20%	57.90%	45.20%	46.30%
All Females	612	61	161	168
	53.80%	42.10%	54.80%	53.70%
**Gender vs Age**				
Male 18-24	47	7	10	12
	4.10%	4.80%	3.40%	3.80%
Male 25-34	130	35	42	47
	11.40%	24.10%	14.30%	15.00%
Male 35-44	136	30	44	48
	12.00%	20.70%	15.00%	15.30%
Male 45-54	108	4	20	20
	9.50%	2.80%	6.80%	6.40%
Male 55-65	104	8	17	18
	9.10%	5.50%	5.80%	5.80%
Female 18-24	78	8	19	21
	6.90%	5.50%	6.50%	6.70%
Female 25-34	135	16	34	35
	11.90%	11.00%	11.60%	11.20%
Female 35-44	155	16	45	47
	13.60%	11.00%	15.30%	15.00%
Female 45-54	144	13	41	42
	12.70%	9.00%	13.90%	13.40%
Female 55-65	100	8	22	23
	8.80%	5.50%	7.50%	7.30%
